# SARS-CoV-2 Psychiatric Sequelae: A Review of Neuroendocrine Mechanisms and Therapeutic Strategies

**DOI:** 10.1093/ijnp/pyab069

**Published:** 2021-10-14

**Authors:** Mary G Hornick, Margaret E Olson, Arun L Jadhav

**Affiliations:** Roosevelt University, College of Science, Health and Pharmacy, Schaumburg, IL, USA

**Keywords:** COVID-19, Neuroendocrine, Neuropsychiatric disorders, Neuroimmunology, Therapeutic strategies

## Abstract

From the earliest days of the COVID-19 pandemic, there have been reports of significant neurological and psychological symptoms following SARS-CoV-2 infection. This narrative review is designed to examine the potential psychoneuroendocrine pathogenic mechanisms by which SARS-CoV-2 elicits psychiatric sequelae, as well as to posit potential pharmacologic strategies to address and reverse these pathologies. Following a brief overview of neurological and psychological sequelae from previous viral pandemics, we address mechanisms by which SARS-CoV-2 could enter or otherwise elicit changes in the CNS. We then examine the hypothesis that COVID-19-induced psychiatric disorders result from challenges to the neuroendocrine system, in particular the hypothalamic-pituitary-adrenal (HPA) stress axis and monoamine synthesis, physiological mechanisms that are only further enhanced by the pandemic-induced social environment of fear, isolation, and socioeconomic pressure. Finally, we evaluate several FDA-approved therapeutics in the context of COVID-19-induced psychoneuroendocrine disorders.

Accepte

## Introduction

Severe Acute Respiratory Syndrome Coronavirus 2 (SARS-CoV-2), the etiological agent of coronavirus disease 2019 (COVID-19), has caused the most severe and wide-reaching pandemic since the 1918 influenza virus. As of September 1, 2021, SARS-CoV-2 has resulted in 218 million infections worldwide that resulted in 4.53 million deaths. While SARS-CoV-2 notably affects the respiratory system, producing a life-threatening pneumonia in critical patients, it is now understood that COVID-19 also has numerous neuropsychiatric manifestations. Several retrospective studies have demonstrated incidence of a wide variety of disorders such as insomnia, depression, suicidal ideation, anxiety, confusion, and consciousness disturbances (Szcześniak et al., 2021).

SARS-CoV-2 is not the first viral infection to exhibit neuropsychological symptomology. Acute and chronic impairments of central nervous system (CNS) function have also been reported for past viral epidemics as highlighted in Table 1 (Valerio et al., 2020).

## Neuropsychological impact of coronaviral outbreaks

Coronaviral outbreaks such as SARS-CoV-1, Middle East Acute Respiratory Syndrome Coronavirus (MERS-CoV) and SARS-CoV-2 share this common history of neuropsychiatric sequelae. The 2003 epidemic of SARS-CoV-1 was associated with seizures, optic neuritis, ischemic stroke, myopathy, and axonal motor neuropathy (Lau et al., 2004; Umapathi et al., 2004; Xu et al., 2005). Moreover, impairments in memory, sleep disturbances, increased levels of stress, depression, anxiety, and post-traumatic stress disorder (PTSD) were observed up to 8 years after infection (Hong et al., 2009; Moldofsky and Patcai, 2011). MERS-CoV patients suffered from seizures, acute disseminated encephalomyelitis, encephalitis, stroke, chronic fatigue, depression, and PTSD symptoms for up to 18 months post infection (Saad et al., 2014; Arabi et al., 2015; Lee et al., 2019; Park et al., 2020). With respect to SARS-CoV-2, reports of associated CNS disorders appeared early on, including cases of anosmia, encephalopathy, delirium, and stroke (Helms et al., 2020; Wu et al., 2020; Dhamoon et al., 2021). Rogers, *et al*, authored a systematic review and meta-analysis of neuropsychiatric presentations of acute and post-illness SARS-CoV-1 and MERS along with the initial reports of acute psychiatric disturbances with SARS-CoV-2 in the summer of 2020, signaling scientists and medical professionals to be prepared for similar outcomes (Rogers et al., 2020). In 72 studies involving a total of 3559 cases, acute illness presented primarily with states of confusion, delirium and anxiety for each of the coronaviruses. Post-illness neuropsychiatric manifestations for SARS-CoV-1 and MERS included a prevalence of depression, insomnia, anxiety, memory impairment and fatigue, all forewarning the potential for analogous outcomes in those who recover from SARS-CoV-2 (Rogers et al., 2020). Indeed, a retrospective cohort study provided evidence for substantial neurological and psychiatric morbidity observable 6 months after COVID-19 infection (Taquet et al., 2021b). Most notably, anxiety, depression, and PTSD have been prevalent co-morbidities in COVID-19 patients (Bo et al., 2021). Beyond confirming the increased risk of a new-onset or reoccurrence of psychiatric sequelae following SARS-CoV-2 infection, one study interestingly noted an increased risk for patients with an existing psychiatric disorder diagnosis for contracting COVID-19 itself (Taquet et al., 2021a). Thus, investigating the theoretical mechanisms behind acute and long-term psychiatric sequelae associated with SARS-CoV-2 may also provide some insight into the risk factors for developing severe infection, not only with coronavirus but other critical or chronic illnesses.

## Mechanisms of SARS-CoV-2 entry into the CNS

### ACE2-mediated cellular entry

As evidenced by the neuropsychological sequelae attributed to COVID-19 infection, SARS-CoV-2 can access the CNS. SARS-CoV-2 infects host cells through the binding of transmembrane serine protease 2 (TMPRSS2)-primed viral spike glycoproteins (S) to angiotensin converting enzyme 2 (ACE2) (Hoffmann et al., 2020; Walls et al., 2020). Upon binding of primed S to ACE2, the viral envelope fuses with the host cell membrane. ACE2 is a metallocarboxypeptidase of the renin-angiotensin-aldosterone system (RAAS) that catalyzes the formation of angiotensin 1-7 from angiotensin II and angiotensin 1-9 from angiotensin I. Angiotensin 1-7, which can also be formed from angiotensin 1-9, has vasodilatory and neuroprotective effects. In addition to the well-documented localization of ACE2 receptors in the respiratory, cardiovascular, and GI tract, ACE2 receptors are also present in neuroendocrine organs including the hypothalamus, pituitary, and adrenal gland (Lv et al., 2017; Zou et al., 2020). The highest expression of ACE2 in the brain was found in the amygdala, pons, and medulla oblongata (Lukiw et al., 2020). Therefore, SARS-CoV infection of neurological and neuroendocrine organs is possible.

### Neuronal and hematogenous routes of CNS entry

Viral entry into the CNS can occur through both neuronal retrograde and hematogenous routes. SARS-CoV-2 is proposed to access the central compartment through the olfactory bulb or the subarachnoid space (Desforges et al., 2019). Once in the subarachnoid space, CSF facilitates viral spread throughout the brain. At this point, SARS-CoV-2 can enter any cells meeting the minimum requirement of ACE2 and TMPRSS2 co-expression. Neuroinvasion through the olfactory system is implicated in a model of SARS-CoV-2 infection in golden Syrian hamsters (Zhang et al., 2020a). After two days of intranasal inoculation, SARS-CoV-2 was identified in olfactory neurons and coupled with the presence of acute inflammation of olfactory epithelium (Bryche et al., 2020). In humans, however, viral antigens have not been detected in the olfactory bulb and ACE2 is not expressed in olfactory system neurons, suggesting that CNS entry is more complex and involves ulterior routes (Hamming et al., 2004; Bryche et al., 2020).

The presence of SARS-CoV-2 RNA in patient stool and the well-documented GI symptoms of infection suggest enteric neuroinvasion is also possible. SARS-Cov-2 may enter the CNS via enteric neurons, including the vagus and splanchnic nerves, which enable viral passage through the gut-brain axis. Histological immunostaining demonstrates both ACE2 and TMPRSS2 expression by the ENS, glial cells and choroid plexus of the small and large intestine, thus meeting the minimum requirements for SARS-CoV-2 viral entry (Deffner et al., 2020). As will be described in later sections, viral activity in the GI tract can have direct CNS consequences in terms of regulating several neurochemicals involved in psychiatric disorders.

There are also pathways for SARS-CoV-2 to enter the CNS through the hematogenous route. Infected leukocytes serving as a viral reservoir can shuttle the virus across the BBB. Additionally, BBB endothelial cells express ACE2, which could mediate viral entry into the CNS (Guo et al., 2008; Baig et al., 2020). Ultimately, irrespective of the method of entry, once in the brain, SARS-CoV-2 is capable of both directly and indirectly inducing a myriad of pathophysiological alterations, any of which may result in psychiatric symptoms. It should be noted, however, despite the detection of SARS-CoV-2 in the brain and CSF, that the levels of virus in the CNS of humans is relatively low, and symptoms have mostly been associated with inflammatory and vascular mechanisms, rather than direct infection of neurons (Solomon, 2021).

## Activation of psychoneuroendocrine mechanisms

It is important to note that neurological and psychological effects of viral pandemics are rooted in two complementary causes: (1) the neuropsychological sequelae resulting directly from pathological mechanisms of viral infection and (2) the psychological consequences of social response to pandemic conditions **(Fig 1)**. Decades of research have demonstrated that viral infection functions as a neurological stressor, which subsequently influences brain function, behavior, and mood. Psychiatric disorders can result from challenge or interference with the neuroendocrine stress systems, in particular the hypothalamic-pituitary-adrenal (HPA) axis and/or monoamine synthesis and reuptake. While this review emphasizes the direct theoretical mechanisms of COVID-19-related psychiatric symptomatology, indirect mechanisms may also contribute. Public health measures including lockdown and quarantine, in addition to widespread fear of infection and increased caregiver responsibilities, induce unprecedented situations of isolation, uncertainty and stress. Anxiety or depressive symptoms could result from negative reactions to the new realities imposed by social isolation in both infected and uninfected people (Zhang et al., 2020b).

## Hypothalamic-Pituitary-Adrenal axis disruption

As a normal response to stress, the steroidal hormone cortisol is a vital anti-inflammatory and immunosuppressive regulator that follows a 24-hour cycle governed by a complex endocrine feedback loop known as the HPA axis (Jones and Gwenin, 2021). Severe acute and chronic stress along with viral infection or trauma can, however, disrupt the HPA axis and cause fluctuations in this natural cycle.

**Cytotoxicity, ACE2 depletion, and hypocortisolism**

There are several mechanisms by which SARS-CoV-2 may disrupt the HPA axis **(Fig 2)**. Previous reports from the SARS-CoV-1 epidemic noted long-lasting (>3 months) central adrenal insufficiency, or hypocortisolism, in up to 40% of patients following recovery with evidence of hypothalamic neuronal degeneration and viral presence (Leow et al., 2005). Additionally, autopsies of SARS patients revealed direct viral infection of adrenal cortical cells leading to necrosis and degeneration (Ding et al., 2004), while more recent autopsies of COVID-19 patients describe microscopic adrenal alterations including hemorrhage, necrosis and adrenalitis (Freire Santana et al., 2020; Hanley et al., 2020). Direct viral cytotoxicity is possible due to the expression of ACE2 receptors in the hypothalamus, pituitary, and adrenal cortex (Lukiw et al., 2020; Steenblock et al., 2020). ACE2 receptors and TMPRSS2 are co-localized in adrenocortical cells of the zona fasciculata and zona reticularis. Entry into these cells and the subsequent depletion of ACE2 may directly interfere with the cortisol synthetic pathway (Mao et al., 2020). Loss of ACE2 in the hypothalamus causes alterations in corticotropin-releasing hormone (CRH), thereby affecting the stress response and anxiety-related behavior. In transgenic animal models, overexpression of ACE2 in the brain leads to decreased anxiety via the reduction of plasma corticosterone, CRH in the hypothalamus, and pituitary-expressed proopiomelanocortin (Wang et al., 2016; Wang et al., 2018; Alenina and Bader, 2019). Conversely, SARS-CoV-2-mediated shedding of ACE2 may contribute to higher anxiety as well as depressive symptoms. Unsurprisingly, disruption of the HPA and the resultant hypocortisolism seen in SARS-CoV patients has been associated with increases in anxiety, depression, and PTSD (Raony et al., 2020). SARS-CoV-1 survivors with HPA dysfunction mostly recovered to normal glucocorticoid levels within one year, while their chronic fatigue and psychiatric morbidities persisted for up to four years (Lam et al., 2009). The chronic fatigue, headaches, and other symptoms of “long-COVID” during the present pandemic indicate that a similar mechanism may exist with SARS-CoV-2.

**ACTH mimicry and secondary adrenal insufficiency**

Beyond direct cytotoxic damage to the hypothalamus, pituitary and/or adrenal glands, coronaviruses are also known to express certain amino acids that mimic adrenocorticotropic hormone (ACTH) (Pal, 2020). This molecular mimicry results in the immune system attacking both the viral amino acids and the host's own ACTH, creating the potential for secondary adrenal insufficiency which could account for CNS presentations without viral presence in the brain itself. One recent study of patients infected with SARS-CoV-2 specifically measured plasma cortisol and ACTH on days 1 and 2 of hospital admission (Alzahrani et al., 2021). Despite the acute illness, 64.3% presented with clinically low cortisol levels (<300 nmol/L), while 82.1% had low-normal levels of ACTH. Of patients tested multiple times (days 1-2, 3-5, and 8-11), 60% were found to have an inadequate cortisol response, the severity of which positively correlated with the severity of COVID symptoms. In another study, it was found that plasma cortisol levels were significantly lower in critically ill COVID patients versus critically ill non-COVID patients (Mao et al., 2020). Approximately 50% of the non-COVID patients presented with elevated cortisol levels, as typically seen during the acute phase of illness or trauma. It is worthy to note that the hypocortisolism seen in SARS coronaviral infections does not only occur during the acute phase, but also post-recovery and even in asymptomatic patients (Chua and Chua, 2021; Hashim et al., 2021). Low levels of cortisol in asymptomatic or mild cases may explain why some of these patients go on to have relatively difficult 'long COVID' symptoms. It would be interesting to compare the post-infectious neuropsychiatric sequelae of asymptomatic/mild COVID patients with and without altered cortisol levels.

**Hypercortisolism and inflammation**

This disruption of the HPA axis may additionally account for the rare but troubling accounts of new-onset psychosis in the post-infectious stage (Haddad et al., 2020; Lorenzo-Villalba et al., 2020; Parra et al., 2020). In the healthy stress response, an increase of cortisol is associated with a decrease in IL-6. In acute psychosis, schizophrenia, SARS-CoV-1 and SARS-CoV-2, however, there is disruption of the glucocorticoid-immune circuits such that both IL-6 and cortisol may be elevated (Raony et al., 2020). Disruption of the HPA axis, increases in pro-inflammatory cytokines, and the exacerbation of social isolation may create the perfect epigenetic storm for patients to experience new-onset psychological pathologies.

**Impaired monoamine synthetic pathways**

In addition to disruptions in the HPA axis, depletion of ACE2 may have a significant effect on monoamine synthesis, which could exacerbate or trigger mental health disorders **(Fig 2)**. ACE2 in the GI tract is responsible for regulating the uptake of amino acids, including tryptophan, the precursor for synthesis of serotonin, dopamine, and melatonin (Hashimoto et al., 2012; Singer et al., 2012). ACE2-deficient mice present with reduced uptake of tryptophan and subsequent reduced serotonin levels (Alenina and Bader, 2019). Indeed, deletion of ACE2 caused a 75% reduction of tryptophan in the blood, resulting in a significant decrease of serotonin in the blood (48%) and brain (32%) (Klempin et al., 2018). One of the main biological functions of ACE2 is to serve as a counterbalance to the ACE-RAAS pathway via the ACE2-angiotensin (1-7)-MAS axis. ACE2 reduces Angiotensin II to Ang 1-7, which then binds to MAS to initiate important signaling pathways essential for the production of nitric oxide (NO), brain-derived neurotropic factor (BDNF), and a host of other anti-inflammatory and neuroprotective functions (Samavati and Uhal, 2020). Thus, the impaired tryptophan uptake and subsequent reduction in serotonin, along with upsetting the balance between ACE/ACE2, could be a potential mechanism by which SARS-CoV-2 impairs both mood and memory.

Tryptophan is also an essential amino acid for the synthesis of dopamine, which plays a critical role in regulating mood and neurogenesis. By use of a multi-experiment matrix to determine correlation links between mRNAs across human microarray datasets, researchers recently determined that the gene most often co-expressed with ACE2 was dopa decarboxylase (DDC) (Nataf, 2020). Importantly, DDC converts L-DOPA into dopamine and L-5-hydroxtryptophan into serotonin. Co-expression of ACE2 and DDC in both intestinal epithelial cells and the brain facilitates the synthesis of dopamine and serotonin, likely through the ACE2-Ang (1-7)-MAS pathway (Pawlak et al., 2001). Depletion of ACE2 and the subsequent imbalance of the ACE/ACE2 metabolic pathways may thus impair both serotonin and dopamine synthesis resulting in higher rates of mental "fog", depression, and anxiety in COVID-19 patients. Indeed, increased plasma ACE and genotypes that promote this imbalance are associated with higher rates of depression, bipolar disorder, and psychotic symptoms, all of which can increase suicidal ideation (Conejero et al., 2021). When combined with HPA axis dysregulation and viral inflammation, the reduction of positive mood-regulating monoamines may create the perfect platform for psychiatric disorders.

Another biochemical pathway deleteriously affected by ACE2-mediated reduction in tryptophan availability is the melatonergic pathway. Decreased tryptophan can alter levels of N-acetylserotonin (NAS), which in turn disrupts the pineal melatonin cycle, lowering levels of BDNF and impairing pineal melatonin regulation of the immune system (Anderson and Reiter, 2020). Additionally, pro-inflammatory cytokines upregulated by viral infection can switch off pineal melatonin production, resulting in the inflammatory system failing to dampen immune cells at night. This inflammation also induces aryl hydrocarbon receptor ligands that further suppress serotonin and melatonin availability

(Anderson and Reiter, 2020). Sleep disturbances are a known symptom of both acute and long COVID. Alterations in the pineal melatonin cycle and serotonin levels, coupled with inadequate sleep, may therefore contribute to both neurological and psychological disturbances in the acute and sub-acute phases of SARS-CoV-2 infection.

**Neuroinflammatory effects on neuroendocrine pathways**

While depletion of ACE2 by SARS-CoV-2 may directly disrupt or inhibit the psychoneuroendocrine pathways related to stress, anxiety and depression, it is additionally important to note the indirect effects on these pathways due to viral-induced neuroinflammation, particularly in light of the relatively minimal amount of virus within the CNS itself. SARS-CoV-2 causes the release of several pro-inflammatory cytokines, most of which are also elevated in psychiatric disturbances. In particular, the virus has been noted to increase the levels of interleukins 2, 6, 1β, and 10, along with tumor necrosis factor alpha (TNF-α) and interferon gamma (IFN-γ) (Raony et al., 2020). High levels of IL-6 have been noted in the blood and/or CSF of patients suffering from depressive disorders, schizophrenia, PTSD, and sleep disorders. Similarly, elevated expressions of IL-2, IL-10, IL-1β, TNF-α, and/or IFN-γ may be detected in depression, schizophrenia, bipolar disorder, PTSD, and suicidal ideation (Black and Miller, 2015; Irwin et al., 2016; Wang and Miller, 2018; Enache et al., 2019; Yang and Jiang, 2020). Neuroinflammation, regardless of the inducing factor, can lead to neurotoxicity and deregulation of several neuronal networks and signaling pathways, including the HPA axis and monoamine synthesis/reuptake **(Fig 2)**.

**Cytokine storm and the HPA axis**

The release of pro-inflammatory cytokines during the acute phase of COVID-19 infection, particularly during a 'cytokine storm' event, can have severe impacts on the HPA axis. These cytokines act on the hypothalamus to increase CRH secretion, the pituitary to secrete ACTH, and the adrenal cortex to release cortisol, dramatically elevating the levels of circulating glucocorticoids that protect against continued non-specific immune activation and immunomodulator release. During an active SARS-CoV-2 infection, a disruption in the negative feedback loop between the HPA axis and the immune system may prevent the glucocorticoid-mediated suppression of the immune system, resulting in hyperactivity of the HPA (Raony et al., 2020). This hyperactivity is further exacerbated by the viral depletion of ACE2 in the hypothalamus, pituitary, and/or adrenal glands (Lukiw et al., 2020; Mao et al., 2020). While hypercortisolism has been noted in cases of acute COVID-19, positively correlated with higher levels of depression and stress (Garg et al., 2020; Ramezani et al., 2020), hypocortisolism is prevalent in the sub-acute phase. In addition to antibody-mediated destruction of ACTH due to molecular mimicry, certain cytokines may also decrease the activity of the HPA axis (Raony et al., 2020). Neuroinflammation plays a significant role in the edema, necrosis, hemorrhage, and degeneration noted in the adrenal cortex, hypothalamus, and pituitary, all of which can contribute to post-infectious sequelae related to hypocortisolism (Garg et al., 2020; Alzahrani et al., 2021; Dolatshahi et al., 2021). Increases in oxidative stress and mitochondrial damage brought on by the viral-induced release of pro-inflammatory cytokines may result in further cytotoxicity and neurodegeneration, presenting as changes in cognition or mood.

**Cytokines and monoamine synthesis**

In addition to targeting the HPA axis, the release of pro-inflammatory cytokines can severely disrupt the monoamine synthetic pathways, potentially worsening or contributing to the onset of post-COVID psychiatric sequelae. Recalling the potentially decreased levels of tryptophan due to SARS-CoV-2 ACE2 depletion, viral-induced pro-inflammatory cytokines can further reduce tryptophan levels, directly impacting serotonin and melatonin synthesis. Specifically, IFN-γ, IL-1β, and TNF-α shift the metabolism of tryptophan from indolamine synthesis (serotonin) towards kynurenine production by upregulating indoleamine 2,3-dioxygenase (Savitz, 2020). This inflammatory induction of the kynurenine pathway has further implications with regards to psychiatric symptoms. Kynurenine is metabolized into either kynurenic acid or quinolinic acid, both of which have been identified as factors in the pathophysiology of mood disorders and psychosis. Quinolinic acid, an NMDA receptor agonist with neurotoxic capabilities, is elevated in patients with depression or suicidal ideation (Erhardt et al., 2013; Meier et al., 2016). Interestingly, the other metabolite, kynurenic acid, is an NMDA antagonist with purported neuroprotective properties that has been found to be elevated in the CSF of patients with schizophrenia and psychosis (Plitman et al., 2017; Sellgren et al., 2021). While the precise role these kynurenine metabolites play in various psychiatric conditions remains to be fully elucidated, neuroinflammation clearly adds to the pathophysiological mechanisms behind psychiatric symptoms of SARS-CoV-2 infection.

**Therapeutic considerations for treating the psychoneuroendocrine symptoms of SARS-CoV-2**

Given the immediate and long-term significance of SARS-CoV-2-induced neuropsychiatric sequelae, we would be remiss in describing their mechanisms without highlighting therapeutic strategies currently under investigation. These potential treatments and their mechanisms are summarized in **Fig 2**.

**Corticosteroids**

The success of dexamethasone in the treatment of COVID-19 provides strong evidence for the ability of SARS-CoV-2 to disrupt the HPA axis. As discussed above, a subset of COVID-19 patients experience hyperinflammation after acute viral infection, affording them a more serious prognosis. Significantly elevated levels of pro-inflammatory cytokines characterize the SARS-CoV-2 cytokine storm and are positively correlated with psychiatric co-morbidities, including depression, psychosis, PTSD and suicidal ideation. The combination of SARS-CoV-2-induced hyperinflammation and sub-acute hypocortisolism prompted investigation into the use of glucocorticoid therapy for COVID-19 treatment. In the Randomized Evaluation of COVID-19 Therapy (RECOVERY) trial (n = 6425), dexamethasone provided a mortality benefit to oxygen and/or ventilation supported patients (Horby et al., 2021). Specifically, deaths were reduced by 20% in oxygen supported patients and 33% in patients receiving mechanical ventilation. Our current understanding of the effects of exogenous glucocorticoid treatment on neuropsychiatric outcomes following critical illness is convoluted, but several studies demonstrate potential benefits (Hill and Spencer-Segal, 2021).

PTSD is a prevalent neuropsychiatric co-morbidity of SARS-CoV-2 infection and is purported to result from hyperinflammation and sub-acute hypocortisolism. In both non-illness and critical illness populations, low cortisol levels have been correlated with PTSD (Delahanty et al., 2000; Meewisse et al., 2007; Yehuda, 2009; Fink, 2011; Morris et al., 2012). In a cohort of ICU patients with acute respiratory distress syndrome (ARDS), patients with lower cortisol levels exhibited higher incidences of PTSD symptomology 6-months post discharge (Spencer-Segal et al., 2017). Genetic polymorphisms in the glucocorticoid receptor (GR) also forecast the vulnerability of a patient to PTSD as homozygous BcII G allele carriers exhibit lower cortisol levels that are inversely correlated to PTSD intensity (Hauer et al., 2011).

Complementary to the relationship between endogenous cortisol and PTSD severity, exogenous corticosteroids, such as dexamethasone, exhibit protective properties against post-ICU psychopathology (Sijbrandij et al., 2015; Kok et al., 2016). Several studies observed lower PTSD incidence with glucocorticoid usage. Namely, use of hydrocortisone reduced PTSD symptomology in patients with septic shock (Schelling et al., 1999; Schelling et al., 2001). A randomized, controlled clinical trial for postsurgical PTSD prevention with hydrocortisone demonstrated significant reductions in PTSD symptoms at 6 months (Schelling et al., 2004). Whether low basal cortisol levels are involved in the pathogenesis of PTSD or only a marker for increased vulnerability is unknown, though this relationship suggests that SARS-CoV-2 related hypocortisolism can increase PTSD risk, especially in hospitalized patients. It should be noted, however, glucocorticoid administration has been found to have little effect on the incidence of anxiety and depression unlike for PTSD (Hauer et al., 2012; Kok et al., 2016; Medici et al., 2017).

**ACE inhibitors (ACEi)**

Given that SARS-CoV-2 appropriates ACE2 for cellular entry, viral infection in patients taking ACEi's for hypertension continues to be of paramount interest and debate. Despite early reports that ACE2 inhibition could promote the upregulation of lung membrane-attached ACE2 and accordingly increase SARS-CoV-2 susceptibility, recent clinical trials conclude that ACEi and ARB use does *not* correlate with increased vulnerability to COVID-19 infection, disease severity, or poorer outcomes (South et al., 2020; Vaduganathan et al., 2020; Asiimwe et al., 2021). In fact, numerous systematic reviews and clinical trials suggest potential benefits. One meta-analysis of 11 studies and 8.4 million patients demonstrated a 13% reduced risk of contracting SARS-CoV-2 in individuals taking an ACEi. Moreover, analysis of 67,644 COVID-19 patients on ACEi treatment exhibited a 24% reduced risk of all-cause mortality (Chu et al., 2021). The potential benefits of ACEi's against COVID-19 are likely specific to hypertensive patients and purported to result from increasing lung membrane ACE2 expression during the second phase of SARS-CoV-2 infection (Huang et al., 2020). While this is the same mechanism that led to questioning continued ACEi/ARB treatment during the acute phase of SARS-CoV-2 infection, enhanced lung membrane ACE2 expression during the sub-acute phase is now hypothesized to prevent COVID-19-induced cytokine storm and reduce ARDS (Gil et al., 2020; McKee et al., 2020).

Combined SARS-CoV-2-mediated decoupling of the HPA axis and immune system, hypocortisolism, and virus-induced downregulation of ACE2 expression has potential implications for the psychoneuroendocrine system, including neuroinflammation, which can produce degeneration and/or changes in cognition, and mood disturbances. As a result, use of ACEi therapy may benefit COVID-19 patients with hypertension co-morbidity that develop neurological and/or psychiatric sequelae. The use of captopril in hypertensive patients has led to antidepressant effects (Zubenko and Nixon, 1984;

Deicken, 1986; Germain and Chouinard, 1988). ACEi's have also demonstrated a reduction in depression in Alzheimer's patients (Isingrini et al., 2009). In addition to depression, ACEi and ARB use has significantly reduced Alzheimer's Disease (AD)-related cognitive decline and led to increased cerebral blood flow (Kume et al., 2012; Zhuang et al., 2016). Few reports evaluating the effects of ACEi and ARB use on COVID-19-induced neurological and psychiatric disorders are available, though a cohort study of 151 senior patients in Japan (mean age 60 ± 19 years) exhibited significantly lower incidence of new-onset or worsening cognitive function in those being actively treated for hypertension at the time of COVID-19 infection (Matsuzawa et al., 2020).

manuscript

**Anti-inflammatory biologics**

Because the most severe presentations of COVID-19 are driven by a hyperinflammatory immune response during the sub-acute phase of infection, anti-inflammatory antibodies have been a subject of investigation for treatment of SARS-CoV-2. Evaluated anti-inflammatory antibodies for COVID-19 treatment have included the anti-IL6 monoclonals tocilizumab and sarilumab, the anti-IL1 antibody anakinra, and monoclonals targeting GM-CSF and TNFα (adalimumab) (Cavalli et al., 2020). While the ability of anti-inflammatory antibodies to reduce COVID-19-related severity and mortality has met mixed results, the defined role of cytokine storm in driving COVID-19-induced psychiatric sequelae prompts a deeper look at these therapeutics. As noted in previous sections, elevated pro-inflammatory cytokines are observed in both COVID-19 and psychiatric disorders. It would therefore make sense that lowering pro-inflammatory cytokines levels would not only reduce the propensity for COVID-19-induced cytokine storm, but also the incidence of associated mood disorders. Several studies have identified significant improvement in depressive symptomology associated with chronic inflammatory conditions (Choy and Calabrese, 2018; Kappelmann et al., 2018; Khandaker et al., 2018). However, a recent study that investigated the effect of anti-IL6 treatment with tocilizumab on depression in the critically ill found significantly higher depression scores 28 days post drug administration (Knight et al., 2021). Depressive scores accounted for the related symptomatology of anxiety, fatigue, sleep, and pain. Interestingly, despite the pharmacological evidence that antagonizing IL-6 would dampen psychiatric manifestation of critical illness, the opposite was observed in the clinic. Similarly, a single case study report of new-onset depression following initiation of anti-IL1β (anakinra) in a patient with rheumatoid arthritis (RA) was reported in 2011 (Jonville-Bera et al., 2011). Despite managing the patient's RA symptoms, depressive symptoms appeared six weeks after therapeutic initiation and escalated to major depressive disorder, suicidal ideation, and inpatient hospitalization after two months. Discontinuation of anakinra and management with antidepressant therapy reduced the depressive symptoms, and depression did not reoccur even following discontinuation of clonazepam and citalopram. More promising results in this arena are observed with adalimumab, a monoclonal antibody with anti-TNFα activity. Adalimumab treatment is correlated with significantly reduced odds of psychosis versus non-immunological therapy, improved psychological functioning in individuals with psoriasis, and significant reductions in depression and anxiety in ankylosing spondylitis (Arısoy et al., 2013; Essali et al., 2019; Leman et al., 2020). Currently, there is a dearth of research investigating the relationship between anti-inflammatory cytokine treatment and the manifestation and/or progression of psychiatric symptoms related to COVID-19. Of importance, due to the complexity of mood disorders, treatment targeting inflammatory mechanisms may require the blockade of multiple cytokines. Significantly more research is required to validate the utility of these repurposed therapeutics in COVID-19 psychoneuroendocrinology.

**Ketamine**

Severe cases of COVID-19 require hospitalization, intensive care intervention and sedation; however, the majority of clinically available sedatives fail to address SARS-CoV-2 cytokine storm. Ketamine, an alternative sedative and anesthetic, reduces depressive symptomology and suicidal ideation by reducing IL-6 and C-reactive protein (CRP) levels (Dale et al., 2012; Weinbroum, 2012; Chen et al., 2018; Corriger and Pickering, 2019; Weinbroum, 2021). Ketamine additionally prevents spikes in post-operative IL-10 and TNFα in comparison to opioid-based analgesia (Welters et al., 2011). Preclinical and clinical studies of ketamine have indicated that it may enact its anti-depressive effects by reducing levels of circulating kynurenine and blocking inflammation-and kynurenine metabolite-induced activation of the NMDA receptor(Steiner et al., 2013; Zanos et al., 2018; Savitz, 2020). Given the incidence of stress-induced mood disorders associated with COVID-19 and ICU stays, ketamine offers an attractive alternative for critical care providers, particularly when considering the mechanisms of action proposed in **Fig 2**.

**Antidepressants**

As discussed, SARS-CoV-2-mediated downregulation of ACE2 can interfere with monoamine synthesis pathways, affecting concentrations of key chemical mediators of mood and behavior. Because mood disorders are associated with elevated levels of pro-inflammatory cytokines that are also elevated in SARS-CoV-2 infection, antidepressants that modulate monoamine levels and demonstrate anti-inflammatory properties may have a role in treating COVID-19-induced psychiatric sequelae. Antidepressants have been shown to attenuate immune activation by lowering levels of pro-inflammatory cytokines with positive effects on mental health (Lu et al., 2017). In the context of COVID-19, fluvoxamine, a selective serotonin reuptake inhibitor (SSRI), has demonstrated the ability to significantly reduce risk of intubation and death in a double blind, randomized, placebo-controlled study, putatively by controlling sepsis-related inflammatory mechanisms (Lenze et al., 2020). Preliminary

Accepted observational studies suggest that this association is also significant for other antidepressants, including fluoxetine, paroxetine, escitalopram, venlafaxine and mirtazapine (Hoertel et al., 2021). Finally, the recent finding that there is no significant difference in the anti-depressive effects of psilocybin and escitalopram in a phase 2, double-blind, randomized, controlled clinical trial in patients with chronic, moderate-to-severe depression highlights an opportunity for psychedelics in long-haul COVID-19 psychiatric disorders (Carhart-Harris et al., 2021; Kelly et al., 2021). As with each of the aforementioned therapeutics, the use of specific antidepressants requires far more research in the context of COVID-19 and its psychiatric sequelae.

**Conclusions**

As the number of SARS-CoV-2 infections continue to rise despite increasing vaccination, it is imperative that researchers and clinicians expand our knowledge of the mechanisms by which COVID-19 affects mental health. Based on the theoretical psychoneuroendocrine mechanisms highlighted in this review, it would be interesting to see future studies evaluate biomarkers such as cortisol, monoamines, and pro-inflammatory cytokines along with the use of established mental health scales in patients infected with SARS-CoV-2. Only through well-designed and thorough study can we determine the actual, rather than theoretical, pathways by which COVID-19 and other conditions with similar post-illness neuropsychiatric sequelae act, and use that knowledge to help not only those affected by COVID-19, but also help prepare us for future pandemics.

**Acknowledgements**

None.

**Statement of Interest**

None.

**Figure Legend**

**Figure 1.** Direct (solid line) and indirect (dashed line) mechanisms by which the COVID-19 pandemic has led to an increase in psychological and mood disorders. * denotes mechanisms discussed in the present manuscript.

**Figure 2.** Potential mechanisms by which SARS-CoV-2 directly (solid lines) and indirectly (dashed lines) alters neuroendocrine pathways leading to psychological and neurological disturbances.

- ❖ Indicates available therapeutic options that are being studied in conjunction with SARS-CoV-2.

Table 1: Summary of Viruses Associated with Neuropsychiatric outcomes

| VIRUS | Neurological and Psychological Sequelae | References |
| --- | --- | --- |
| CHIKUNGUNYA | Anxiety<br>Depression<br>Chronic Fatigue<br>Disseminated Encephalomyelitis<br>Guillan-Barre Syndrome (GBS)<br>Optic Neuropathy | (Bhatia et al., 2015; Cerny et al., 2017) |
| INFLUENZA A (H3N3, H1N1) | Encephalopathy<br>Febrile Seizures<br>Ischemic Stroke<br>Meningitis | (Toovey, 2008; Okusaga et al., 2011) |

| | | |
|---|---|---|
| | Mood Disorders | |
| POLIOVIRUS | Meningitis<br>Psychiatric Disturbances | (Nielsen et al., 2007; Lo and Robinson, 2018) |
| WEST NILE | Encephalitis<br>Meningitis<br>New-Onset Depression | (Kramer et al., 2007; Murray et al., 2007) |
| ZIKA | Behavioral and Memory Deficits<br>Guillain–Barré Syndromes (GBS)<br>Microcephaly | (de Araújo et al., 2016; Musso et al., 2019; Raper et al., 2020) |

Figure 1

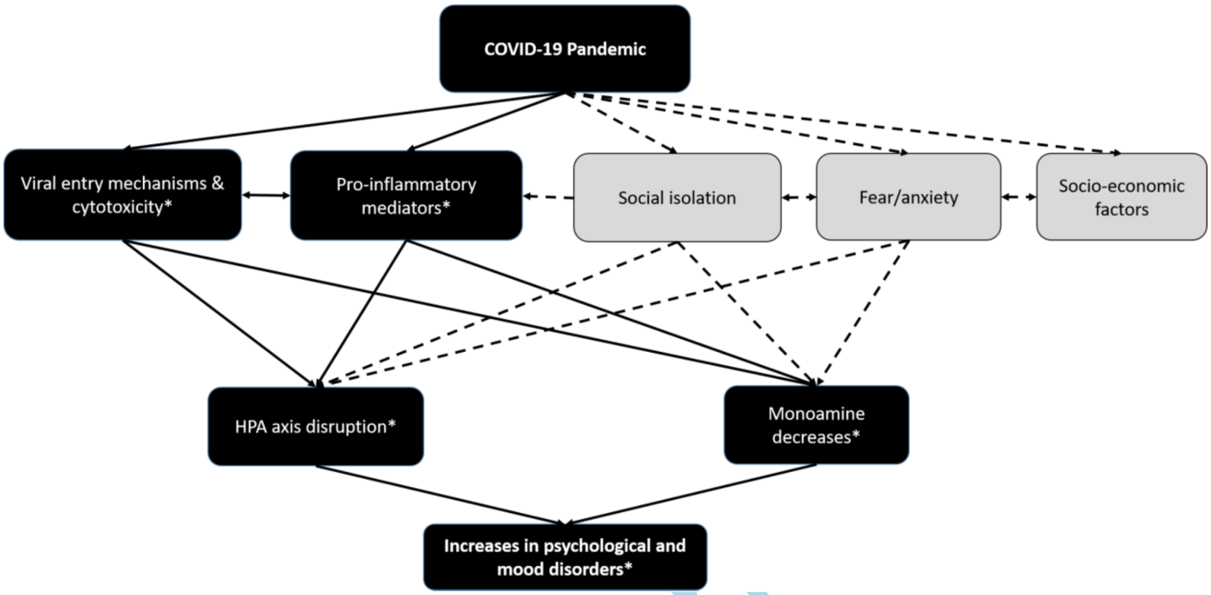

Accepted Man

Figure 2

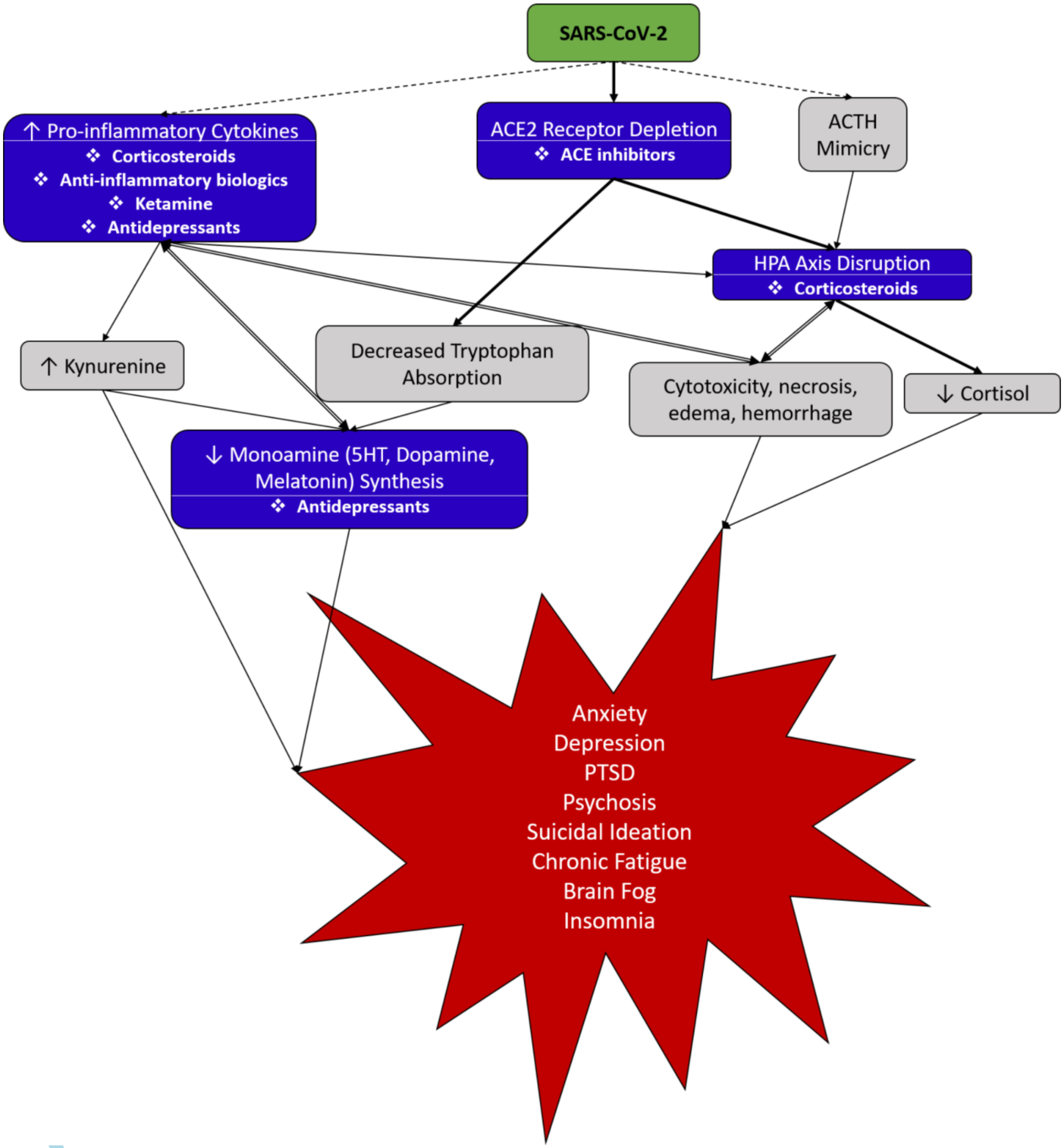

SARS-CoV-2
↑ Pro-inflammatory Cytokines
❖ Corticosteroids
❖ Anti-inflammatory biologics
❖ Ketamine
❖ Antidepressants
ACE2 Receptor Depletion
❖ ACE inhibitors
ACTH Mimicry
HPA Axis Disruption
❖ Corticosteroids
↑ Kynurenine
Decreased Tryptophan Absorption
Cytotoxicity, necrosis, edema, hemorrhage
↓ Cortisol
↓ Monoamine (5HT, Dopamine, Melatonin) Synthesis
❖ Antidepressants
Anxiety
Depression
PTSD
Psychosis
Suicidal Ideation
Chronic Fatigue
Brain Fog
Insomnia
